# Metabolic Syndrome: A Narrative Review from the Oxidative Stress to the Management of Related Diseases

**DOI:** 10.3390/antiox12122091

**Published:** 2023-12-08

**Authors:** Giovanni Martemucci, Giuseppe Fracchiolla, Marilena Muraglia, Roberta Tardugno, Roberta Savina Dibenedetto, Angela Gabriella D’Alessandro

**Affiliations:** 1 Department of Agricultural and Environmental Sciences, University of Bari Aldo Moro, 70126 Bari, Italy; gmartem@libero.it; 2 Department of Pharmacy–Drug Sciences, University of Bari Aldo Moro, 70126 Bari, Italy; marilena.muraglia@uniba.it (M.M.); roberta.tardugno@uniba.it (R.T.); roberta.dibenedetto@uniba.it (R.S.D.); 3 Department of Soil, Plant and Food Sciences, University of Bari Aldo Moro, 70126 Bari, Italy; angelagabriella.dalessandro@uniba.it

**Keywords:** metabolic syndrome, diabesity, gut microbiota, dysbiosis, cardiovascular diseases, neurodegeneration

## Abstract

Metabolic syndrome (MS) is a growing disorder affecting thousands of people worldwide, especially in industrialised countries, increasing mortality. Oxidative stress, hyperglycaemia, insulin resistance, inflammation, dysbiosis, abdominal obesity, atherogenic dyslipidaemia and hypertension are important factors linked to MS clusters of different pathologies, such as diabesity, cardiovascular diseases and neurological disorders. All biochemical changes observed in MS, such as dysregulation in the glucose and lipid metabolism, immune response, endothelial cell function and intestinal microbiota, promote pathological bridges between metabolic syndrome, diabesity and cardiovascular and neurodegenerative disorders. This review aims to summarise metabolic syndrome’s involvement in diabesity and highlight the link between MS and cardiovascular and neurological diseases. A better understanding of MS could promote a novel strategic approach to reduce MS comorbidities.

## 1. Introduction

Metabolic syndrome (MS) is one of the most common health problems today, affecting almost 30% of the world’s population. It is considered one of the major issues of industrialisation in developing countries and will affect more than half of the population in the next 20 years [1]. The increase in MS is linked to many factors, such as sedentary lifestyle, environmental factors and diet as the Food Away from Home (FAFH), constituting an important public health problem [2]. It has been estimated that MS [3], especially among women [4,5]. Approximately 24% of adults in the USA, 12–37% of the Asian population and 12–26% of the European population suffer from this disease [6], and about 44% of people are in the age group ≥ 50 years. The concept of MS was introduced in the 1920s [7]. MS has been described as a cluster of cardiometabolic risk factors, including hyperglycaemia [8,9], central obesity (waist circumference), hyperinsulinemia and insulin resistance (IR) [10], hypertension, hypertriglyceridaemia, low plasma high-density lipoprotein (HDL) and high cholesterol levels. Moreover, ageing and hormonal changes have been associated with the development of MS [11,12,13,14,15]. Other pathological disorders closely correlated to MS include liver diseases, such as non-alcoholic fatty liver disease (NAFLD), non-alcoholic steatohepatitis (NASH) [16], neurological diseases [17] and cancer [18].

Different clinical criteria have been adopted for the definition of MS by international organisations (Table 1).

According to a joint agreement between international organisations, individuals suffering from MS must show three clinical signs on the following five criteria: central obesity (specific definition in relation to the population and country), TG ≥ 150 mg/dL and/or on pharmacological treatment; HDL-C < 40 mg/dL in males and <50 mg/dL in females; diastolic BP ≥ 130 and systolic ≥ 85 mmHg and/or under pharmacological treatment; and FBS ≥ 100 mg/dL and/or drug treatment [20].

The aim of this work is to provide an overview of the main risk factors of MS, related to oxidative stress, diabesity, cardiovascular diseases and neurological diseases to support potential strategic approaches to solve the complications of MS.

## 2. Free Radicals, Oxidative Stress and Metabolic Syndrome

Free radicals are produced during cell metabolism and redox processes. They include reactive oxygen species (ROS), reactive nitrogen species (RNS) and reactive sulphur species (RSS) [22,23]. All free radicals are involved in body pathophysiological processes [24]. Superoxide can damage molecules (DNA, proteins and lipids) [25]. The hydroxyl radical reacts strongly with most organic and inorganic molecules (DNA, proteins, lipids, amino acids, sugars, vitamins and metals) faster than its speed of generation [26]. It is estimated that OH• is responsible for 60–70% of the tissue damage caused by ionising radiation [27]. Hydroxyl radicals are involved in several disorders, such as cardiovascular diseases [28] and cancer [29]. Nitric oxide is also involved in many physiological processes, such as neurotransmission, relaxation of smooth muscle, vasodilation and regulation of blood pressure, gene expression, defence mechanisms, cell function and regulation of inflammatory and immune mechanisms, as well as in pathological processes such as neurodegenerative disorders and heart diseases [30]. Cells and the body can protect themselves from free radicals through antioxidants to lower the concentration of free radicals and maintain redox homeostasis in the body [31]. The antioxidant defence systems consist of endogenous (generated in situ) and exogenous antioxidants (supplied through foods). They play the role of neutralising excess free radicals and protecting cells from their toxic effects, helping to prevent diseases. When body defence mechanisms are reduced, free radicals, generated by endogenous and exogenous sources, can cause direct oxidative damage to biological molecules and organs, with consequent oxidative stress and metabolic disorders [24].

Oxidative stress concerns intracellular damage as well as secondary damage due to the cytotoxic and mutagenic characteristics of the metabolites produced [24,32,33]. In particular, carbon reactive compounds, such as malondialdehyde (MDA) and 4-hydroxynonenal (4-HNE), which are formed during lipid oxidation and carbohydrate glycoxidation, reacting with cell tissues and proteins generate the advanced lipid peroxidation end-product (ALE) and the advanced glycation end-products (AGE), which cause protein-level dysfunction, such as loss of activity and increased sensitivity to proteases [34] and in inflammatory responses and apoptosis [35]. As a result, oxidative stress contributes significantly to the pathogenesis of different diseases [31]. Carbon reactive compounds, such as MDA, 4-HNE or oxidised LDL, have been found in cardiovascular disease [36], atherosclerosis [37], diabetes [38], obesity and IR [39]. The role of oxidative stress in MS is rapidly evolving as a result of evidence and related manifestations, including atherosclerosis, hypertension and T2D [40], low-grade inflammation [41], adiposity and IR [42,43], cardiovascular diseases and neurological disorders [17].

## 3. Metabolic Syndrome and Diabesity

### 3.1. Diabetes Mellitus

Diabetes is a complex syndrome characterised by hyperglycaemia induced by an altered secretion of insulin or by a poor insulin action when the pancreas does not produce enough insulin or when the body cannot effectively use the insulin it produces, or both cases. There are different types of diabetes: type 1, type 2, gestational diabetes and secondary or other specific types of diabetes [44].

Type 1 diabetes (T1D) accounts for about 5% of all types of diabetes [45]. It is a disease especially prevalent among young people, which causes the destruction of insulin-producing pancreatic cells and the lack of insulin [46]. T1D is associated with autoimmunity against pancreatic beta cells, i.e., the destruction of beta cells caused by the expression of autoantibodies against insulin (IAA), antibodies against insular cells (ICA), antibodies associated with insulinoma protein-2 antibodies (IA-2A), glutamic acid decarboxylase antibodies (GADA) and zinc transporter antibodies 8 (ZnT8A) [47]. Type 2 diabetes (T2D) is more common in older and overweight people [48,49]. T2D includes most diabetic individuals worldwide (90–95%) [45]. The development from pre-diabetes to T2D is more gradual and prolonged than T1D [50]. Gestational diabetes affects 3–9% of pregnant women, mainly during the second or third trimester, due to insufficient insulin secretion [51]. Patients with gestational diabetes, over time, may experience a high risk of developing permanent T2D [52,53]. Secondary types of diabetes include several specific causes, such as beta cell function from monogenic defects, pancreatic diseases and drug/chemical-induced endocrinopathies [44].

Clinical diabetes trials have been linked to high blood glucose levels observed during fasting (≥126 mg/dL) and 2 h after an oral glucose load (≥200 mg/dL). A recent diagnostic test of diabetes, which reflects glycaemia, is the measurement of glycated hemoglobin (Alc ≥ 6.5% (≥48 mmol/mol) [54]. Clinically, recognizable changes in carbohydrate metabolism that are characteristic of diabetes occur when blood sugar levels reach levels that cause glycosuria and polyuria, leading to polydipsia. Patients with T2D may not show these clinical signs early due to the gradual increase in blood sugar over time. The use of glucose-based diagnostic tests can lead to an increased risk of retinopathy. Autoantibodies to IAA, ICA, IA-2A and ZnT8A are employed in the diagnosis and prediction of T1D in both children and adults, and they can differentiate between latent autoimmune diabetes in adults (LADA) and T2D [47,55,56].

Hyperglycaemia is a key component of MS. A high concentration of glucose in the blood contributes to oxidative stress through several mechanisms, such as glucose auto-oxidation, the formation of advanced glycated products (AGEs) and the increased oxidation of arachidonic acid (ARA) [57]. Intense glucose variation has many side effects modifying the redox balance, increasing circulating free fatty acids (FFAs), NADPH oxidase activity and TNF-α [58]. A change of the first sites in the mitochondrial membrane leads to the activation of complex II [59] and to the excessive formation of O_2_•^−^ by a loss of electrons [60]. NADPH oxidase (NOX) producing ROS is the main source of production of glucose-induced ROS in the vascular system, kidneys [61], liver [62] and β-cells [63]. The network of glycation reactions that produce oxidative stress along with glucose toxicity is important [64]. Advanced glycation end-products (AGE) promote inflammation by interacting with AGE receptors (RAGE) in immune system cells [65]. The activation of RAGE stimulates intracellular signals, including kinase (MAP kinase, PI3 kinase), and transcription factors, such as nuclear factor-kB (Nf-kb) and activator protein-1, which further activate the expression of cytokines, chemokines, enzymes and growth factors, resulting in a pro-inflammatory environment that leads to oxidative stress [66]. Hyperglycaemia induces an increase in the production of free radicals that alter both enzymatic and non-enzymatic antioxidant defence [67]. For example, the accumulation of sorbitol, resulting from enzymatic conversion for excessive glucose, disrupts osmotic balance [68], and the formation of AGEs induced by increased fructose production causes β-cell injury [69] and peripheral IR [70]. High levels of AGEs represent a high risk factor for T1D [71] and lead to the development from pre-diabetes to diabetes [72]. Haemoglobin A1c, due to the glycation of haemoglobin, is the most important biomarker of glycaemic exposure [73] related to oxidative stress. The increase in Hb1Ac predicts the risk of microvascular complications in T1D [74] and cardiovascular disease in T2D [75].

Insulin resistance is one of the most important mechanisms linked to MS [14,76]; it is complex and not perfectly defined. The insulin hormone plays an important role in the regulation of glucose concentration, lipid homeostasis and energy storage [76]. It promotes the preservation of glucose as glycogen in the liver and skeletal muscles, and the deposition of fatty acids as triglycerides in adipose tissue [77]. During insulin resistance, anabolic metabolism is inhibited by reducing the absorption of glucose and the conservation of glucose as glycogen and triglycerides, while increasing the hydrolysis of the triglycerides stored and their mobilisation as free fatty acids and glycerol, and the liver increases the production of glucose through gluconeogenesis and the inhibition of glycogen synthesis and depot.

IR promotes the breakdown of energy substrates, such as glucose in the brain, foetal immune system, fat in the organs and compensatory hyperinsulinaemia [77]; thus, its metabolic negative effects could be considered a mechanism activated in some physiological conditions, such as stress inflammation and fasting, and while in the chronic state, it constitutes the symptoms of the MS [78]. The increased production of ROS/RNS is a trigger for IR in different animal models [79]. When cells fail to compensate for IR through increased insulin production, a reduced glucose tolerance occurs and the excessive production of mitochondrial ROS causes intracellular oxidative stress, which can damage cellular macromolecules as well as inactivate or modulate the insulin receptor and its substrate function [40].

IR appears to precede and predict the development of diabetes mellitus. The molecular mechanism of IR is linked to an increase in FFAs, triacylglycerol (TAG), diacylglycerol (DAG), acylcarnitine and ceramide [80,81], which are associated with the accumulation of lipids in the liver involved in IR and T2D. A higher FFA concentration results in an increase in intracellular glucose concentration and a reduction of muscle glucose consumption, which may lead to a reduction in glucose transport and insulin receptor signalling [82]. Different processes are associated with IR.

Free radicals and oxidative stress play an important role in the dysfunction of β-cells in diabetes mellitus, as they are involved in the disruption of pancreatic β-cell proliferation through the alteration of cell cycle regulators, with the resulting development and progression of diabetes [83]. Metabolic body homeostasis is strictly controlled by the release of the insulin hormone from the pancreatic β-cells, which interact with target tissues, such as muscle, adipose tissue and the liver, to eliminate excess glucose from the blood. On the contrary, glucagon released from pancreatic-α cells mobilises glucose from these tissues during periods of fasting or increased energy expenditure to maintain the blood glucose concentration at a constant level. The alteration of these metabolic processes leads to chronic conditions of hyperglycaemia and dyslipidaemia, or glucolipotoxic conditions, which have a harmful influence on the pancreatic islets, causing manifestations of metabolic diabetes syndrome [83].

Oxidative stress due to progressive mitochondrial and metabolic dysfunction derived from elevated glucose and/or fatty acid oxidation can reduce insulin secretion from β-cells as well as impair insulin signalling in target tissues. Low levels of antioxidant enzymes in β-cells, such as glutathione peroxidase, catalase, thioredoxin and superoxide dismutase, determine the accumulation of free radicals in β-cells, especially in diabetes [84]. β-cell dysfunction can induce high superoxide anion (O_2_•^−^) and hydrogen peroxide (H_2_O_2_) production [85], with the consequent alteration of mitochondrial function, reduction of ATP production and insulin secretion [67]. H_2_O_2_ and O_2_•^−^ influence the secretion of glucose-stimulated insulin [48], and H_2_O_2_ may inhibit β-cell metabolic activity and the secretion of insulin [86]. The limited antioxidant defence system of β-cells can lead to the initiation or amplification of the inflammatory response [87]. β-cell dysfunction and apoptosis depend on complex gene activation, regulated by transcription factors such as NF-κB [88], which translates into the nucleus and stimulates the transcription of several genes related to pro-inflammatory responses. Gene 88 and kinase associated with interleukin-1 receptor (IL-1) have been shown to be involved in homeostasis loss, tissue damage and the start of T1D [89,90].

Inflammation stimulates the development of IR and diabetes through a complex mechanism that involves different kinases and signalling pathways. Inflammation is linked with increased cytokine levels, such as IL-6, IL-1β IL-10 IL-17, tumour necrosis factor α (TNF-α) and interferon γ (IFN-γ) created by immune cells in the islets [91]. T1D can be considered an autoimmune disease with its attack on pancreatic β-cells, leaving intact the α-cells and δ-cells of the islets [46]. A common mechanism linking T1D and T2D may be the activation of NOX and the resulting production of free radicals. NOX is stimulated by glucose, saturated fatty acids, endocrine factors and pro-inflammatory cytokines [85], such as TNF-α, IFN-γ and IL-1β, involved in the disorder and decay of β-cells in T1D, and in inflammation associated with T2D [92]. Inflammatory condition plays an important role in the development of TD1 and TD2 [48,93]. Free radicals created by the inflammation of tissues or immune cells by interacting with the insulin receptor adversely affect its response [48,94]; as a result, the amount of insulin produced by the beta cells of the pancreas becomes inadequate, causing resistance to insulin, the fundamental key to T2D disease. There is a relationship between obesity, inflammation and IR. Weight gain and obesity are linked to IR and to a chronic low-grade inflammatory condition defined as meta-inflammation [95,96,97]. During IR, metabolic processes are altered. Adipose tissue and skeletal muscles reduce the uptake and storage of glucose as glycogen and triglycerides. Conversely, this increases the hydrolysis of stored triglycerides and their mobilisation as free fatty acids and glycerol, and the liver increases glucose production through gluconeogenesis and inhibits the synthesis and storage of glycogen [77]. An excessive intake of nutrients can induce oxidative stress in adipose tissue [98] and cause the dysregulation of the function of adipocytes, which is manifested by the inhibition of the differentiation of adipocytes, a higher infiltration of immune cells in adipocytes and an increase in the secretion of peptides and proteins defined as adipocytokines [99]. Then, the phenotypic passage to the inflammatory macrophage occurs [100], as well as the increased infiltration of macrophages into the adipose tissue [96,101] and the induction of chronic inflammation into the white adipose tissue. It has also been proposed that with the enlargement of adipocytes, the blood supply becomes insufficient, and hypoxia occurs. This leads to cellular necrosis and T-cells and macrophages infiltrating the adipose tissue, causing an overproduction of adipocytokine, such as retinol-binding protein-4 (RBP-4), TNF-α, IL-6, IL-8 and monocyte chemoattractant protein-1 (MCP-1) [102,103]. In addition to causing chronic inflammation in adipocytes, cytokines are also released into the bloodstream, and eventually, the process becomes systemic [96] by inhibiting insulin signalling, resulting in insulin resistance [104]. Therefore, chronic inflammation in obesity plays a key role in the pathogenesis of IR, which is recognised as a symptom of MS [78].

IR and hypertension are constituents of MS. Clinical observations indicate that about 50% of people with hypertension tend to show hyperinsulinemia or glucose intolerance, while about 80% of patients with T2D have hypertension [105,106]. Patients with essential hypertension are more intolerant to glucose and resistant to insulin. The compensatory hyperinsulinaemia that prevents T2D in insulin-resistant people acts in the kidneys and the sympathetic nervous system, improves the reabsorption of sodium and increases the sympathetic tone contributing to an increase in blood pressure [107]. The loss of water and the deprivation of sodium due to insufficient sodium intake or excessive sodium loss may activate both the renin–angiotensin–aldosterone and the neuro–endocrine systems to increase blood pressure [108,109].

Insulin may have vascular-protective or deleterious effects. IR through an altered nitric-oxide oxidative mechanism can influence compensatory hyperinsulinaemia, which can activate the MAPK [76], resulting in higher values of vasoconstriction, proinflammation, sodium, water retention and blood pressure [110]. Studies in rats have shown a link between oxidative stress and activated oxidative stress-associated inflammation with an increase in blood pressure, vascular dysfunction and IR [110]. Ultimately, a significant increase in the risk of the occurrence of cardiovascular diseases and T2DM derives from the coexistence of IR and hypertension [105].

### 3.2. Diabesity

**Figure 1:**
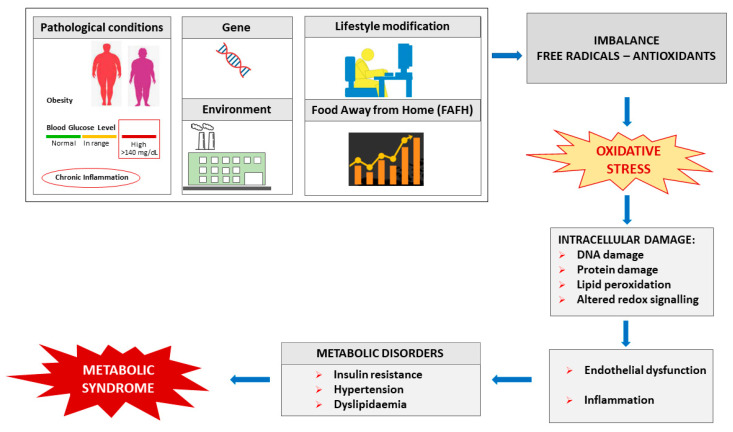
Metabolic syndrome (MS) related processes. Under pathophysiological conditions, the imbalance between free radicals/oxidants and antioxidant defences leads to oxidative stress. The resulting oxidative stress causes intracellular damage (DNA, lipids, proteins) and redox alteration, inducing the irreversible accumulation of oxidation products that promote endothelial dysfunction, which leads to insulin resistance, hypertension, dyslipidaemia and, ultimately, MS.

**Figure 2:**
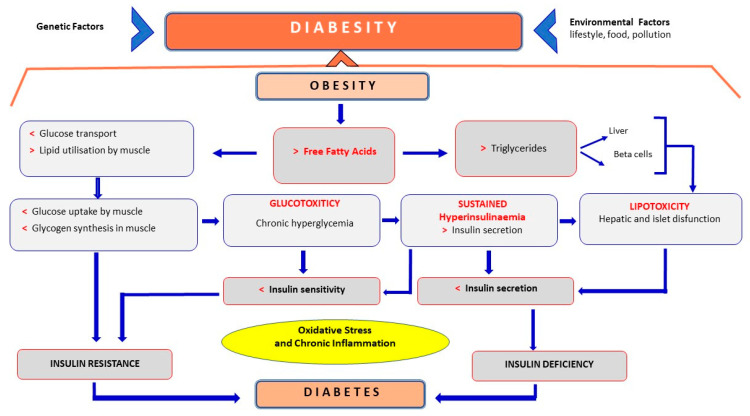
Pathophysiology of diabesity. Genetic and environmental factors (lifestyles) regulate the function of the pancreatic islets and the interaction between diabetes and obesity. Hyperglycaemia, resulting from reduced insulin sensitivity due to the reduction of the functional mass of β-cells, is closely related to obesity, which plays a crucial role in oxidative stress and inflammatory metabolic processes, glucotoxicity and lipotoxicity, and is associated with insulin resistance/deficiency.

Diabesity is considered a multifactorial pathophysiology (Figure 1) involving genetic and environmental factors. The World Health Organisation (WHO) underlines the similarity of the trends in obesity and diabetes due to a close relationship between obesity and diabetes. Severe obesity is a major risk factor for the development of type 2 diabetes mellitus (T2DM). The strong association between obesity and diabetes has led to the coining of the term “diabesity”. The majority of people with T2DM are obese, highlighting the central role of increased adiposity as a risk factor for diabetes [111]. Approximately 60–90% of all patients with T2DM are estimated to be obese (BMI ≥ 30 kg/m^2^) or overweight (BMI ≥ 25 kg/m^2^) [112]. Obesity (BMI ≥ 30 kg/m^2^) and an unfavourable lifestyle were associated with a higher risk of T2DM [113]. It has been estimated that for every gain of a kilogram of body weight, the risk of diabetes increases by about 9% [114]. Changing lifestyles, combined with high levels of industrialisation and advances in agricultural and food systems, have led to increased food availability and changes in food consumption patterns, which are the co-factors responsible for the spread of obesity and diabetes among the young adult population [115]. Figure 2 summarises the main steps involved in obesity and diabetes and the close link between the two syndromes responsible for the onset of diabetes. Obesity results in an increase in plasma levels of FFAs derived from meals and lipolysis of adipose tissue [116], which affects IR [117,118]. In addition, visceral obesity fat being more metabolically and lipolytically active releases more FFAs into the bloodstream [119], increasing their cellular uptake and subsequent mitochondrial β-oxidation; this inhibits glucose metabolism at the level of substrate competition [116].

The increase in FFA levels stimulates the liver and skeletal muscles towards greater oxidation of FFAs for energy production, resulting in a reduction in their ability to absorb and metabolise glucose. The prevalent use of lipids at the expense of glucose leads to a reduction of glucose uptake and glycogen synthesis rate in skeletal muscles [120]; this induces a state of chronic hyperglycaemia (glucotoxicity) that further impairs insulin sensitivity [121]. The increase of fat in and around the liver, skeletal muscle and pancreatic β-cells results in the elevated mitochondrial production of toxic reactive lipid species that cause oxidative damage, inflammation and cellular dysfunction. The release of FFAs then induces lipotoxicity as lipids and their metabolites create oxidative stress on the endoplasmic reticulum and mitochondria; this causes insulin-receptor dysfunction and a consequent insulin-resistant state. The increase of toxic metabolites in the β-cells of the pancreatic islet decreases insulin production and increases β-cell apoptosis, accelerating the development of diabetes [116,118]. Therefore, hyperinsulinaemia constitutes the mechanism by which pancreatic β-cells initially compensate for the deterioration of peripheral insulin sensitivity, ensuring normal glucose tolerance; when beta cells can no longer compensate, T2DM develops. Peripheral IR in muscles and fat reduces cellular glucose absorption, while IR in the liver results in an inability to suppress glucose production and gluconeogenesis.

Oxidative stress and inflammation also participate in the progression from IR to diabetes. Chronic inflammation, a characteristic of obesity, leads to IR and T2D [122,123].

Literature studies highlight the role of genetic factors that also play a role in diabetes. Genetic factors affect BMI [113,124,125,126], and parental obesity is a risk factor for obesity in offspring [127]. Whole genome studies have identified nearly 150 genetic variants that are associated with body size or obesity risk [128]. However, the combined contribution of all known variants associated with body size measurements is <5%; therefore, the influence of genetic factors on body size remains undefined [129]. However, it is estimated that known genes preach only 15% of T2D and 5% of obesity risk [130,131]. Most T2D genes appear to be related to β-cell dysfunction, with much less involvement in IR-related pathways regardless of obesity [132,133].

Among the environmental factors (Figure 2), lifestyle, such as lack of physical activity, and inadequate nutrition, such as a high-calorie diet and/or prolonged abundance of food, contribute to the manifestation of obesity [134,135].

The increase in T2DM is due to a significant increase in obesity and high consumption of food with high levels of fat and refined carbohydrates [136]. The inability of adipose tissue to remove free fatty acids from blood circulation has been reported to contribute to the onset and development of MS [137]. In addition, the increase in the plasma levels of adipokines (leptin, plasminogen activator-inhibitor 1), cytokines (TNF-α, resistin and IL-6) and non-esterified fatty acids in obese individuals may block the action of insulin and promote metabolic dysfunction [138].

There is a link between dietary sugars and body weight gain, likely due to the excess energy associated with sugar intake [139]. A link was also observed between weight gain and sugar consumption in the form of sugar-sweetened beverages and total energy intake [140]. Fructose, the sweetest of all carbohydrates, is commonly used commercially in soft drinks, juices and bakery products. The consumption of fructose is considered accountable in MS as a lipogenic compound associated with an excessive accumulation of ectopic fat, particularly in the liver [141,142]. Dietary fructose is currently suspected to play a significant role in the development of non-alcoholic fatty liver disease (NAFLD) associated with obesity [142,143]. The mechanisms responsible seem to involve stimulation of hepatic de novo lipogenesis and impaired extrahepatic triglyceride-rich lipoprotein clearance [144].

Non-alcoholic fatty liver disease (NAFLD), renamed metabolic associated fatty liver disease (MAFLD), also includes different conditions, such as steatosis without inflammation (NAFL), the necroinflammatory form of non-alcoholic steatohepatitis (NASH) and the cryptogenic cirrhosis [145,146]. Mechanisms of NAFLD implicate high levels of fatty acids from one’s diet and adipose tissue, high intrahepatic de novo lipogenesis and defective export as very low-density lipoproteins (VLDL) [147]. They are associated with the context of increased IR and systemic low-grade “metabolic” inflammation due to increased visceral adiposity and the release of several pro-inflammatory adipocytokines [148,149,150]. NAFLD is associated with MS, T2D, obesity and cardiovascular disease [149,151], and affects about 30% of the Western population [152]. Mendrick et al. [153] reported that mitochondrial dysfunction also seems to play a role in MS and increases as the disease progresses from IR to T2D, and from non-alcoholic fatty liver disease to non-alcoholic steatohepatitis.

Some studies show that a diet rich in fructose increases the concentrations of intrahepatic fat [154], while other trials suggest that the increases in intrahepatic fat may be more related to the intake of excess energy than that of fructose [155]. In addition, with the reduction in sugar intake in overweight and obese subjects, a decrease in the concentration of intrahepatic fat was observed [156,157]. Several studies have shown a link between sugar intake and the risk of diabetes [158], IR [159], NAFLD [160], hyperuricaemia [161], hypertension [162] and coronary and cardiovascular diseases [163]. Therefore, the French National Agency for Food Environment and Labour Safety has proposed a maximum limit of 100 g/day for the intake of total sugars containing fructose; taking into account the promotion of fruit and vegetables, fruit provides about 40 g of sugars, and vegetables 6 g of sugars.

Overweight and obesity affects a wide range of the population worldwide, from teenagers to the elderly. Different mechanisms are involved in the development of obesity. BMI is used to classify overweight and obesity in adults and is a measure of the risk factor for weight gain. The interaction among genetic factors, energy intake and energy expenditure leads to the accumulation of fat. Overweight and obesity are caused by a chronic imbalance between energy intake and energy expenditure, leading to an increase in fat mass, with a consequent increase in weight. Epidemiological studies up until 2016 have shown a 40% increase in BMI (≥25 kg/m^2^) in adults (21% in men and 24% in women). The morbidity rate of obesity (BMI ≥ 30 kg/m^2^) in men is growing faster than that of women (3–12% vs. 7–16%, respectively) [164]. It has been suggested that high fat intake increases the production of ROS, contributing to MS and diabesity. A diet rich in fat can reduce the enzymatic activity of SOD [165] and GPx and increase the ratio of glutathione/oxidised glutathione (GSH/GSSG), associated with hypertriglyceridaemia and the mitochondrial production of ROS [166]. Lower enzymatic activity of GPx, SOD and CAT was observed in patients with MS, associated with an increase in oxidative stress and a pro-inflammatory state, and was also associated with an increase in BMI and waist circumference, strengthening the establishment of a pro-oxidant condition in obese subjects [167].

Obesity increases with the spread of fat in the body. Fat is classified as brown and white adipose tissue, which can be subcutaneous and visceral. Lipid overflow, also known as adipose tissue expandability, is characterised by the abnormal increase in ectopic lipid reserves at the level of skeletal muscle, the liver and the pancreas. Subcutaneous adipose tissue is the normal fat deposit and is considered less harmful for lipid storage. It can expand by increasing the cell size (hypertrophic obesity) or by increasing the number of cells (hyperplastic obesity). When the nutrient load exceeds the expansion capacity of adipose tissue, lipids accumulate in other tissues (skeletal muscle, the liver and the pancreas) [90,168]. Hypertrophic obesity is linked to an increased risk of T2D [169,170]. In addition, lipid storage in less suitable tissues is associated with increased IR and T2D [168,171] due to the lipotoxicity of lipid metabolites, such as diacylglycerols and ceramides, which hinder insulin signal transduction [172,173,174]. Many scholars believe that T2D basically derives from a disorder of lipid metabolism, rather than from an abnormal glucose metabolism.

The heterogeneity of adipose tissue contributes to the determination of the effects observed in disorders linked to excess fat (overweight/obesity) or fat loss (lipodystrophy) and consequent metabolic risks [175,176]. Several studies have highlighted the dynamic nature of adipose tissue [176], which includes turnover and differentiation [177,178]. The dynamics of adipocyte and lipid turnover regulate the size of fat mass and functionality. Regardless of obesity and age, about 10% of adipocytes are replaced each year; the half-life of the adipocyte triglycerides is about 1.6 years [177]. The production of adipocytes is higher in hyperplastic obesity compared to hypertrophic obesity [179]. The hypertrophy of subcutaneous adipose tissue appears to be associated with glucometabolic disorders, while in visceral adipose tissue, it is more closely associated with dyslipidaemia [180]. A low turnover rate and, therefore, the generation of a few but large fat cells represent an early defect in the development of T2D. Therefore, an altered differentiation of the preadipocytes into adipocytes in the adipose tissue of subjects with abdominal obesity is an important risk factor for diabetes [181]. As previously mentioned, the turnover of adipose cells is high (10%/year), while the lipid turnover is modified six times in 10 years (average lifespan of an adipose cell) [182,183]. A low lipid turnover rate was found in the visceral region of subjects with pronounced obesity [183,184]. Visceral adipose tissue is metabolically more active than subcutaneous tissue, with a greater sensitivity to lipolysis stimulation and a reduced insulin response [184,185].

Visceral obesity plays a key role in metabolic disorders affecting metabolism and inflammatory response [186]. Excess adipose tissue produces different pro-inflammatory cytokines that lead to a state of chronic subclinical inflammation associated with both IR and T2D [187]. Adipocytes produce many pro-inflammatory markers, such as IL-6, IL-1β, C-reactive protein (CRP), resistin, visfatin and TNF-α, whose levels are high in obese patients [96,188]. Three main sites have been implicated as initiators of inflammation in MS: the liver, intestines and fat deposits [189,190,191,192]. Common triggers, such as responses to metabolic stress to chronic caloric excess and consequent cell death can trigger inflammation at each of these sites [193,194,195]. The release of inflammation mediators from one site promotes inflammation in other tissues, amplifying a chronic inflammatory state and generalised tissue dysfunction/damage [189]. The increased secretion of inflammatory cytokines derived from adipocytes is associated with reduced insulin sensitivity in obesity [196]. However, inflammatory processes do not fully explain the development of IR because no changes in inflammatory markers have been detected in subjects with T2D [197]. Thus, inflammation alone cannot be associated with sensitivity to insulin because only a portion of obese subjects develop T2D. The accumulation of fat in the organs and the increase of the pro-inflammatory adipokine circulation leads to multiple disorders, such as diabetes [198], cardiovascular diseases [199] and neurodegenerative conditions [200]. Obese people display an increased development of visceral adipose tissue, which acts as an endocrine organ, secreting many hormones/cytokines and FFAs that are able to affect different functions of tissues [201,202,203]; biological processes, including glucose and lipid metabolism; food intake; inflammation; coagulation; and the maintenance of metabolic homeostasis [202]. In obesity, the expansion of fat reserves induces the dysfunction of endocrine factors, resulting in the metabolic alteration of insulin in the target tissues and in the pancreatic β-cells.

Many adipokines have been characterised by their role in the modulation of energy homeostasis and food behaviour [204], such as leptin and adiponectin. Leptin (16-kDa peptide) regulates food intake and body weight through the central mechanism of the appetite and the peripheral effects on the modulation of energy expenditure [205]. The range of interaction processes includes inhibiting insulin secretion from pancreatic β-cells and glucose utilisation [206]. Changes in the plasma levels of leptin or insulin indicate a state of altered energy homeostasis and adiposity. The brain responds to these changes by regulating food intake to restore the mass of adipose tissue to a normal level [207,208]. In obesity, leptin and insulin levels are elevated due to increased fat mass and IR [17]. Leptin improves glucose homeostasis by decreasing the accumulation of intracellular lipids in the liver and skeletal muscle [209], and by direct activation of AMP-activated protein kinase (AMPK) in skeletal muscle [210]. In obese subjects, hyperleptinaemia associated with leptin resistance contributes to the development of obesity and metabolic disorders. Obese subjects show a strong correlation between leptinaemia and body weight, body fat percentage, BMI and IR [211,212].

Adiponectin secreted by adipocytes is involved in controlling food intake and is closely related to diseases such as obesity and T2D [213,214], in which the biosynthesis of adiponectin is impaired by various inflammatory and oxidative stress factors [215,216]. Adiponectin regulates the metabolic processes of lipids and glucose. It controls glucose metabolism and insulin sensitivity [217], increasing the oxidation of FA, reducing TG in skeletal muscles and the liver and suppressing glucose production in the liver [218]. The levels of adiponectin are paradoxically inversely correlated with body weight or body mass index [219]. In several studies, an inverse relationship was observed between the levels of adiponectin with blood pressure, total cholesterol and low-density lipoproteins (LDLs) [220]. In addition, low hormone concentrations were observed in diabetes, obesity and coronary heart disease [221]. Adiponectin concentrations are strongly related to insulin sensitivity [222], resulting in reduced concentrations in obese patients, obese patients with mild diabetes and obese patients with T2D [222,223,224]. It has been indicated as a predictive marker of T2D in obese subjects even years before the onset of the disease [225]. In obese subjects, other adipokines with higher levels than normal subjects were found, such as resistin [226], visfatin [227], apelin [228], fatty acid-binding protein specific for adipocytes [229], adipsin [230] and irisin [231].

Ghrelin is another orexigenic hormone that plays a role in energy metabolism, stimulating food intake and favouring the increase of weight and fat and plays significant roles as a regulator of glycaemia and insulinaemia [232]. The acylated ghrelin shows hyperglycaemic effects leading to IR, while non-acylated ghrelin contrasts hyperglycaemia and improves insulin sensitivity [233]. High circulating levels of active ghrelin were observed in obese and T2D individuals [234].

### 3.3. Gut Microbiota Interactions

**Figure 3:**
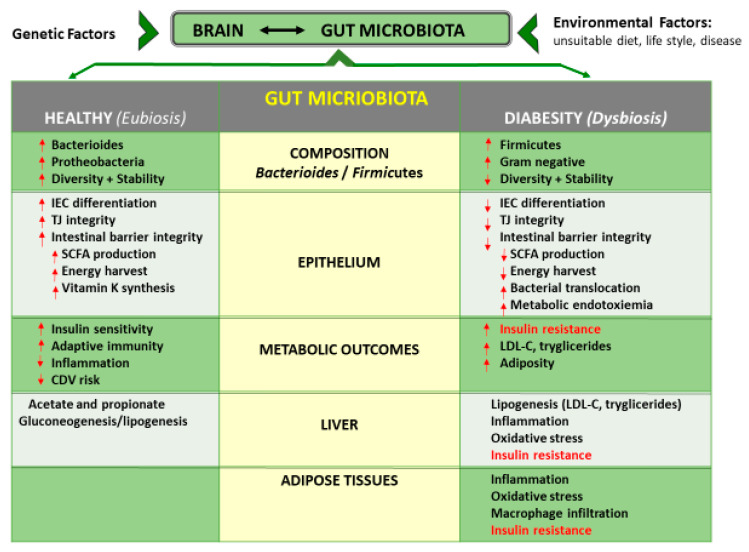
Influence of the gut microbiota on diabesity. Under normal conditions (eubiosis), the gut is dominated by non-pathogenic microorganisms that are important for physiological interactions with other systems, such as the brain and organs and tissues involved in metabolism, to prevent and combat the progression of metabolic syndrome. Dysbiosis in the gut microbiota, caused by many factors (antibiotics, diet, lifestyle), is associated with metabolic alterations leading to diabetes and related comorbidities. CVD, cardiovascular disease; IEC, intestinal epithelial cell; LPS, lipopolysaccharide; SCFA, short chain fatty acid; TJ, tight junction; LDL-C, low-density lipoprotein cholesterol.

Gut microbiota has multiple functional characteristics that result in a wide range of physiological and pathological effects (Figure 3).

Several studies indicate that the gut microbiota is involved in the development of obesity, diabetes and associated comorbidities [235,236,237]. The complex interactions between genetic background and biological and lifestyle factors, influenced by an obesogenic environment, can induce pathophysiological alterations with characteristics of MS and susceptibility to obesity and diabetes, as depicted in Figure 3. The synergistic effect between the gut microbiota and the host involves the bi-directional gut–brain axis, which is of fundamental importance for the regulation of energy metabolism and health [238,239,240]. The symbiotic relationship with the host ensures the adequate development of the metabolic system, performing important functions of health, such as nutritional status and immunity [241,242]. The human gastrointestinal tract (GIT) involves a wide group of micro-organisms (microbiota), including bacteria (dominant) [243], fungi, archaea and viruses [244], which generate a biomass of 1–2 kg, while their combined genomes (microbiome) are about 150 times higher than the human genome, with an estimated 3.3 million microbial genes [245,246,247]. The human genome consists of about 23,000 genes [248]. In recent years, the sequencing of the ribosomal RNA gene (16S RNA) has been considered the most suitable technique for highlighting the diversity and wealth of the microbiome [249,250], and metagenomic sequencing is considered a powerful tool for the analysis of complex microbial communities [251].

The composition of the intestinal microbiota is exclusive for each individual and widely variable in the species, while gene profiles among healthy people are similar [245,252]. The intestine is sterile at birth, although colonisation of the intestine seems to begin before birth [253]. Intestinal colonisation occurs during the first months of life and is completed at 3–4 years of age [254]. The developing microbiota is affected by vital events and the infant’s diet [255,256]. A profile of the intestinal microbiota of a newborn at term, delivered vaginally and breastfed, with a balanced maternal milk microbiota, is considered optimal [257].

Infants born vaginally exhibit a microbiota composed of *Lactobacillus*, *Prevotella* and *Sneathia* spp. coming from the vaginal tract, while children born by caesarean section highlight *Staphylococcus*, *Corynebacterium* and *Propionibacterium* spp. [258]. During breastfeeding, *Actinobacteria* predominate among the genus *Bifidobacterium*. The major changes in the intestinal microbiota in the baby occur with the introduction of solid foods and the end of breastfeeding/weaning. At this time, the microbiota acquires new strains, influenced by changes in diet and diseases, and gradually begins to resemble the adult composition [259]. In the human gastrointestinal tract, large groups of bacteria or phyla belonging to *Firmicutes*, *Bacteroidetes*, *Actinobacteria*, *Proteobacteria* and *Verrucomicrobia* were identified, with the two predominant phyla *Bacteroidetes* (Gram-negative) and *Firmicutes* (Gram-positive) accounting for 90% of the gut microbiota [254,260]. There is considerable diversity of species and their numbers [258]. *Firmicutes* phylum contains over 274 genera, including *Clostridium*, *Bacillus*, *Lactobacillus* and *Ruminococcus*, with the genus *Clostridium* as the most representative, as well as the butyrate producers *Eubacterium*, *Fecalibacterium* and *Roseburia*. Bacteroidetes comprise about 20 genera; they are efficient degraders of dietary fiber and include *Bacteroides*, *Prevotella* and *Xylanibacter*, and the most representative is *Bacteroides* [260]. *Bifidobacterium* is an important genus within *Actinobacteria*; *Proteobacteria* includes *Escherichia* and *Desulfovibrio*, while *Verrucomicrobia* includes the genus *Akkermansia*, which degrades mucus [261].

The digestive system, along with its length, is characterised by a different composition of microbiota [242,262]. There is a small diversity and a low amount of microorganisms in the stomach and a large variety and a high number of microorganisms in the large intestine. Several populations of obligatory and optional anaerobic microorganisms act in the degradation of undigested food [263] in an increasing gradient, from stomach to jejunum, ileum and colon [264], and there is continuously increasing diversity and microbial density from the stomach and duodenum (101–103 bacteria/g) to the jejunum and ileum (104–107 bacteria/g), which culminates in the colon (1011–1012 bacteria/g) [242,247]. The small intestine exhibits less microbial diversity compared to the colon due to higher acidity, higher levels of oxygen, shorter transit time and antimicrobial factors mediated by immune cells. The profile of the jejunum is closely related to that of the stomach with the presence of *Bacilli*, mainly of the species *Streptococcaceae* (50–70%) [265,266]; in the ileum and distal ileum, the species *Bacilli* fall, respectively, to 20 and 5% [266]. Conversely, *Clostridia* species, such as IX, XIVb, XIVa and IV, reach 30% of the microbiome, and *Bacteroidetes* species reach 49% [265,266]. The microbial composition of the gut is usually based on the analysis of faecal material, which is easily accessible but does not fully reflect the microbiota content throughout the digestive system. In addition, there is a large inter-individual difference in microflora [267]; thus, the accurate composition of microflora and its functions can be fallacious [268].

Microbiota and their metabolites play a critical role in regulating metabolic pathways in health and disease. The gut microbiota both directly and indirectly performs multiple vital functions for the maintenance of the human host health, including nutrition and metabolism, such as the digestion of complex dietary foods, the production of vitamins, the biotransformation of bile acids, the resistance against infections, the regulation of intestinal permeability and the development of immune cells [269,270]. The microbiota can digest complex plant polysaccharides in one’s diet [271]. Many species belonging to the phyla *Bacteroidetes* and *Firmicutes* possess enzymes, which determine the extraction of energy from the diet. The *Bacteroides* enterotype has a wide saccharolytic potential, and *Ruminococcus* carries and degrades the constituent sugars [272]. Bacterial glycosides hydrolase degrade soluble fibers into short chain fatty acids (SCFAs), such as acetate, butyrate and propionate [273,274]. They are present in a molar ratio of about 60:20:20 [275] and constitute about 5–10% of the energy source in healthy hosts [276]. The major producers of SCFAs include *Roseburia* spp., *Eubacterium rectale* and *F. prausnitzii* and *Clostridium* groups IV and XIVa in the gut [272]. The total amount of SCFA was found to be lower in normal healthy subjects [277].

The fermentation of indigestible carbohydrates occurs especially in the proximal colon, resulting in the production of gas, SCFAs and succinate. In the distal colon, carbohydrates are gradually exhausted, especially in Western diets characterised by low amounts of indigestible carbohydrates; there is a shift in the fermentation of microbial proteins, which produces a diverse range of metabolites, such as branched-chain fatty acids (BCFAs), phenolic compounds, amines and ammonia [278]. Metabolites such as indole and hydrogen sulphide can positively affect the functionality of the intestine and peripheral tissue [279]. SCFAs are readily absorbed by colonocytes [280] and used as respiratory fuels in the preferential order of butyrate > propionate > acetate. While butyrate provides energy to the epithelial cells of the colon, acetate and propionate reach the liver and peripheral organs, where they are used as substrates for gluconeogenesis and lipogenesis. Additionally, acetate is also a substrate for cholesterol biosynthesis [281,282]. The decrease in pH due to SCFAs affects microbial ecosystems [283]; it can inhibit the undesirable growth of microorganisms and increase the absorption of certain nutrients, contributing to the health of the host [284]. SCFAs are important regulators of gut barrier integrity and metabolism. SCFAs, particularly butyrate, play a role in controlling the integrity of the gut epithelial barrier through the regulation of tight junction proteins, which regulate the molecular transit between the lumen and the liver portal system. Butyrate appears to improve intestinal barrier function by increasing the expression of Claudin-1 and Zonula occludens-1, as well as occludin redistribution [285]. Gut microbiota prevents bacteria invasion by maintaining the intestinal epithelium integrity [286] through butyrate producers such as *Faecalibacterium* and *Roseburia*, which increase mucin production, tight junction assembly and mucin degraders *Prevotella* and *Akkermansia* [287]. *Bacteroides vulgatus* and *B. dorei* have been shown to control the expression of tight junction genes in the colon, reducing intestinal permeability, LPS production and endotoxaemia [288]. Butyrate, produced by *Faecali* bacterium *Roseburia*, reduces intestinal permeability through serotonin transporters and peroxisome proliferator-activated receptor gamma pathways (PPAR-γ) [289]. *Akkermansia muciniphila* protects the intestinal mucosa by increasing levels of anti-inflammatory endocannabinoids, which control the intestinal barrier [290]; it was proposed as an indicator of gut integrity [287].

SCFAs from the intestinal microflora have a beneficial effect on host metabolism and appetite. SCFAs play a potential role in controlling glucose and lipid metabolism and energy homeostasis [291,292]. Both the central nervous system (CNS) and the enteric nervous system (ENS) interact with the microbiota (Figure 2) to regulate the metabolism of nutrients, controlling the functions of the GIT and eating behaviour [238,240]. Through vagal neurons, the gut microbiota can control intestinal peptides secreted by enteroendocrine cells, such as cholecystokinin, ghrelin, leptin, tyrosine peptide (PYY) and glucagon-like peptide-1 (GLP-1). The link of SCFAs with G-protein-coupled receptors 43 (Gpr 43) and 41 (Gpr 41) [293] increases plasma levels of satiety hormones derived from enteroendocrine cells, glucagon-like peptide 1 (GLP-1) and peptide YY (PYY) [294,295], as well as leptin from human adipocytes [296], resulting in improved glucose homeostasis and reduced appetite [297]. In particular, by stimulating the activity of peptide YY, Gpr41 increases the intestinal transit rate and reduces the collection of energy from one’s diet [298]. Gpr43 stimulates GLP-1 to increase insulin sensitivity [292], while the activation of Gpr43 on adipocytes suppresses insulin signalling and inhibits fat accumulation in adipose tissue [299].

Propionate and butyrate can prevent IR by the induction of intestinal gluconeogenesis; butyrate through a cAMP-dependent mechanism, propionate by the intestinal circuit-neuronal brain involving Gpr41 and the consequent release of glucose into the portal vein leads to the regulation of blood glucose and insulin sensitivity [300]. Overall, the upregulation of intestinal gluconeogenesis results in a reduction in hepatic glucose production, contributing to improved energy homeostasis.

SCFAs have different functions in tissues [301,302]. They prevent obesity by inhibiting the action of lipoprotein lipase, which determines the accumulation of triglycerides in adipocytes [303]. The effects of SCFAs on energy expenditure are associated with the regulation of the genes PPARγ co-activator 1α (PPARGC1A, encoding PGC1α) and uncoupling protein 1 (UCP1) in brown adipose tissue [304]. Butyrate triggers the oxidation and thermogenesis of fatty acids through increased phosphorylation of the 1α-coactivator of the gamma receptor activated by the peroxisome proliferator (PGC-1α), AMPK in the liver and muscles and expression of PGC-1α and mitochondrial-1 decoupling protein (UCP-1) in brown adipose tissues [305]. Butyrate and propionate also stimulate intestinal gluconeogenesis through the gut–brain circuit, contributing to glucose regulation and metabolic benefits on body weight [300]. Acetate reduces appetite by activating the tricarboxylic acid cycle (TCA), thus altering the expression of the neuropeptide that regulates hypothalamic appetite [306].

Acetate and propionate can increase energy expenditure and lipid oxidation by increasing thermogenesis in adipose tissue and inducing browning of adipose tissue [307], preventing adiposity.

The gut microbiota regulates both the synthesis of bile acids and cholesterol metabolism [308]. Primary bile acids, such as colic acid and chenodeoxycholic acid, are synthesised in the liver from cholesterol [309] and conjugated with glycine or taurine before secretion into bile, which is then deposited in the gallbladder. They are released in the small intestine to support the digestion and absorption of fats, triglycerides, cholesterol and fat-soluble vitamins. Approximately 95% of bile acids are reabsorbed in the ileum, and through the enterohepatic circulation, it returns to the liver at a different time of day for secretion, thus maintaining the gluco-lipidic and energetic homeostasis and preventing hyperglycaemia, dyslipidaemia, obesity and metabolic and cardiovascular disorders [310]. A low percentage of bile acids that escape ileal absorption can be reabsorbed in the colon or modified by intestinal microorganisms to produce secondary bile acids, contributing to pool heterogeneity of bile acids [311,312], and are subsequently absorbed by passive diffusion in the colon and returned to the liver [313]. Secondary bile acids entering circulation act as signals to influence the host’s metabolism [314,315].

Bile acids also have an endocrine function by signalling and activating receptors such as farnesoid receptor x (FXR) and bile acid receptor 1 coupled to proteins G (TGR5), which regulate lipid and glucose metabolism in the liver [316,317]. The activation of the G protein TGR5 receptor on enteroendocrine cells leads to the secretion of GLP-1 [318], which regulates appetite, insulin sensitivity and glucose metabolism [319]. Then, bile acid signalling is associated with the secretion of gastrointestinal hormones PYY and GLP-1, important in the maintenance of energetic and metabolic homeostasis [319,320,321].

Microbial stability reduces obesity through the increased expression and/or activity of fasting-induced adipose factor (FIAF), which regulates lipoprotein lipase produced by the intestine, liver and adipose tissue [322,323], and controls the synthesis of angiopoietin-like factor IV, thus regulating peripheral fat storage and adiposity [324]. The metabolic pathway of antimicrobial peptide (AMP)-activated protein kinase (AMPK) also protects against obesity [325]. The gut microbiota influences both the innate and adaptive immune systems; it plays an important role in the development of CD4^+^ T-cells [326,327]. The association of specific bacterial species with the development of T-cell subtypes has been revealed. For instance, *Bacteroides fragilis* has been shown to induce the development of a systemic Th1 response through its polysaccharide A molecules [328,329], demonstrating that colonic Tregs have a unique TCR repertoire that primarily recognises bacteria from colonic contents. Gut microbiota and its products regulate the development and function of immune cells [269,330]. Butyrate stimulates the formation of peripheral regulatory T-cells [331,332]. The effect on the immune system is obtained through the interaction between microbial patterns, such as lipopolysaccharide (LPS), lipoteichoic acids of bacterial walls, flagellin, stranded RNA/DNA and toll-like receptors (TLRs) of epithelial cells of the intestinal tract [330,333]. For example, TLR5 is a pattern recognition receptor for flagellin, and signals derived from TLR5 are important for the maintenance of intestinal homeostasis [334].

Many factors can affect the composition and/or functionality of the gut microbiota, also known as dysbiosis, including genetics [335], age [336], mode of delivery at birth [256], method of feeding in infants [255], sex [337], geographical location [338], diet [339,340], BMI [341], physical activity [342], antibiotic consumption [343,344] and diseases [345,346].

There is no consensus on the composition of the intestinal microbiota in an obese/overweight individual’s gut [347]. However, obesity-related dysbiosis has been strongly correlated with a higher ratio of *Firmicutes*/*Bacteroidetes* [260], as observed in children [348,349], obese women with MS [350] or obese individuals [351]. Different studies have observed the link between obesity and unbalanced dominant intestinal phyla, with reductions in *Bacteroidetes* associated with a proportional increase in *Firmicutes* [352,353], whereas other studies have found no differences between *Firmicutes* and *Bacteroidetes* in obese individuals [354,355]. Among the phyla *Bacteroidetes* and *Firmicutes*, there are species that promote a higher extraction of energy from the diet and the preservation of extra calories [303]. Jumpertz et al. [355] reported an increase of 20% in *Firmicutes* and a decrease of 20% in *Bacteroidetes* associated with the supplementary extraction of 150 Kcal per day from one’s diet. Many studies show high changes at family, genus and species levels, with a tendency towards excessive growth of bacteria that are more efficient in extracting energy from food, inducing excessive fat accumulation with consequent obesogenic and pro-inflammatory profiles [356,357,358]. The *Firmicutes* phylum contains many species that produce butyrate, which may contribute to increased energy conservation in obese people [355]. Considering obesity due to unbalanced nutrition and inflammatory disease, dysbiosis plays a key role in the development of adiposity and T2D [274]. The gut microbiota plays a crucial role in metabolism, influencing energy balance, glucose metabolism and low-grade inflammation associated with obesity [359]. Among the environmental factors, dietary habits can affect gut microbiota composition. The evolutionary process of the human diet indicates a link with the diet of monkeys, whose genetic diversity is about 2–3% [360]. The ancestral human diet was essentially vegetarian [361], consisting of complex carbohydrates fermented by the intestinal microbiota to produce energy [362]. Western diets are generally low in fiber and rich in fat and digestible sugars [363], as well as high uptake of SFA [364,365], which can lead to an alteration in the composition of the gut microbiota, obesity and diabetes [365]. In particular, the consumption of SFA has been linked to a decrease in *Bacteroides*, *Prevotella*, *Lactobacillus* ssp. and *Bifidobacterium* spp. [366,367,368].

The intestinal microbiota is involved in storing energy through different mechanisms. Intestinal dysbiosis alters the production of gastrointestinal factors related to satiety and metabolism, with a consequent increase in fat storage. The total amount of SCFAs was found to be higher in obese subjects [277]. In animals subjected to a high-fat diet, acetate and propionate cause the expansion of adipose tissue through the inhibition of lipolysis and adipocyte differentiation [369,370]. Metabolite SCFAs can promote the accumulation of fat through the activation of specific receptors (GPR43 and 41), expressed in different cell types (immune cells, endocrine cells, adipocytes) [293]. The activation of GPR43 and 41 is associated with an increased expression of GLP-1 (mechanism involving GPR43) and peptide YY (PYY track, GPR41) in the intestine [282]. Both peptides are related to the reduction of hunger and appetite, but the PYY also reduces intestinal transit and may increase the absorption of nutrients, including SCFAs [371], favouring the increase in weight and obesity. Other microbial messengers to induce obesity involve farnesoid X receptor, proliferator-activated receptor-α [372,373], methylamine N-oxide [374,375] and indoles [375].

The intestinal microbiota can modify the primary bile acids through the activity of the bile salt hydrolase in secondary bile acids (deoxycholic acid, lithocholic acid, ursodeoxycholic acid) and modify their bioactivity and bioavailability [376,377], influencing the metabolic responses involved [378]. Modified bile acids contribute to their pool heterogeneity [311]. Intestinal bacteria, by altering the composition of bile acids, can modify the cellular metabolism and the physiology of the host [376,379]. Secondary bile acids derived from the microbiota enter the circulation and, as signalling molecules, influence the host’s metabolism [315]. Bile acids interact with receptors such as FXR and TGR5, affecting cardiovascular function [380]. Secondary bile acids are involved in pathological processes, including irritable bowel syndrome (IBS), colon cancer [381], liver problems [382], gallstones and high cholesterol in some patients [383].

Bacterial activities on the bile acids may also affect the activation of the receptor G protein TGR5 on enteroendocrine L cells, which leads to the secretion of GLP-1, regulating appetite, insulin sensitivity and glucose metabolism [319].

Fat storage can also be influenced by FIAF, which regulates LPL [323]. Microbial suppression of the FIAF, a peptide potent inhibitor of LPL, promotes obesity [322,323], as well as the major synthesis of angiopoietin-like factor IV [324].

Changes in the composition and activity of the gut microbiota affect type 1 diabetes. The genus *Bacteroides* is the most representative of dysbiosis associated with T1D in children [287,384,385], known as juvenile diabetes. In addition, it is characterised by an increased *Bacteroidetes*–*Firmicutes* ratio [287]; large amounts of *Blautia* spp., *Streptococcus* spp. and *Rikenellaceae*; and low levels of *Lactobacillus*, *Bifidobacterium*, *Blautia coccoides*, *Faecalibacterium* spp. and bacteria that degrade mucin as *Akkermansia* spp. and *Prevotella* spp. [287,386]. It should be noted that, although T1D is one of the most represented chronic diseases in childhood, about 25% of adults report the disease [387], the incidence of which has been increasing for several decades in Western countries after the Second World War [388,389]. The onset of T1D was associated with a change in the *Bacteroidetes*:*Firmicutes* ratio [287,390]. Then, the *Bacteroidetes*:*Firmicutes* ratio, along with the *Bacteroides dorei* and *B. vulgatus*, have been proposed as predictors of T1D-associated autoimmunity [391].

T1D is regarded as a disease characterised by insulin deficiency resulting from autoimmune destruction or loss of the function of pancreatic β-cells [392]. Autoimmunity against insulin is expressed through the release of cytokines and chemokines by beta cells that attract lymphocytes and macrophages, causing immune invasion of the islets. Circulating autoantibodies targeting autoantigens, such as insulin, as well as cytokines released by infiltrating lymphocytes, such as IL-1β, IFN-γ and TNF-α, cause progressive destruction of β-cells [393,394]. However, the triggering factor for autoimmunity in T1D is not well defined [395].

The methods of birth and feeding of the infant influence the composition of the microbiota. In particular, children born vaginally show a higher level of bacterioides and a quicker maturation of the microbiota [396]. Breast milk contains favourable properties against T1D [397]. *Bifidobacterium* and *Lactobacillus*, transferred from mother to child [398,399], preserve the intestinal barrier, stimulating the production of IgA antibodies, and are involved in the production of SCFAs [398,400]. *Bifidobacterium infantis* plays an important role in immune regulation [401] and in the protection of children at risk of T1D. Bacteroides species, such as *B. dorei*, inhibit immune stimulation and inflammatory cytokines modulated by high levels of LPS derived from *Bacteroides* [402]. The link between LPS and TD1 involves toll-like receptor-3 and innate immune signal transduction adaptor (MyD88) in mice [402,403].

A high-fiber diet is linked to a butyrate beach SCFAs [404]. Butyrate plays an important role in modulating the immune response through the differentiation of regulatory T lymphocytes (Tregs), such as Fox3p + Tregs, and through the inhibition of inflammatory cytokines, such as IFN-γ [332,405].

The SCFA-producing species, such as *Bifidobacterium adolescentis*, *Roseburia faecis* and *Faecalibacterium prausnitzii*, are capable of producing butyrate, generate fewer autoantibodies [406] and are protective in children at risk of T1D. This occurs with various clusters of *Clostridium* able to form butyrate from acetate, along with the *Bifidobacterium* species that can form butyrate through lactate metabolism. This substrate can be transformed into butyrate or in other SCFAs, such as acetate, succinate and propionate, during their anaerobic bacterial fermentation in the gut, depending on the type of microbiota [407]. It has also been reported that butyrate producers, such as *Faecalibacterium* and *Roseburia,* and mucin degraders *Prevotella* and *Akkermansia* perform a protective action against T1D [287,365,386,390,406] because butyrate increases mucin synthesis, tight joint assembly and epithelial cell integrity [408]. In contrast, when microorganisms such as *Bacteroides* and *Veillonella* are abundant, the substrate follows the pathway for succinate, acetate and propionate, which alter mucin synthesis, tight junctions and paracellular permeability [407]. Thus, dysbiosis can trigger a low-grade chronic inflammation state and exposure to LPS, which, by binding to TLR4 and its co-receptors, stimulate a cascade of responses, ultimately determining the release of pro-inflammatory molecules that interfere with the modulation of glucose and insulin metabolism [406].

Dysbiosis triggers pathogenic mechanisms that promote the development of obesity, T2D and MS [282,409]. However, the composition and/or metabolic activity of intestinal microorganisms contributing to the onset of obesity and T2D remain unclear.

Distinct modifications in the gut microbiota have been associated with T2D [410]: a noticeable decline in the phylum *Firmicutes* and *Bifidobacterium* spp., a positive correlation between plasma glucose and the relationship between *Bacteroidetes* and *Firmicutes*, the relationship between *Bacteroides-Prevotella* and *Clostridium coccoides* and the content of *Betaproteobacteria*. Thus, an increased community of Gram-negative bacteria (*Bacteroides-Prevotella* and *Betaproteobacteria*) is related to glucose intolerance [410], and the *Faecalibacterium prausnitziis* species has been negatively correlated with an inflammatory state and diabetes [411].

Gut microbiota in patients with T2D mellitus is characterised by the depletion of different bacteria that produce butyrate, such as the species *Clostridium*, *Eubacterium rectale*, *Faecalibacterium prausnitzii*, *Roseburia intestinalis* and *Roseburia inulinivorans*, and by an increase in opportunistic pathogens [412,413]. The correlation between changes in the gut microbiota and T2D markers indicates that *Clostridium* species are negatively related to fasting glucose levels, glycated haemoglobin (Hba1c) and insulin [413]. A high concentration of glucose in the blood may be expected from a reduction in the proportion of anaerobic species, in particular, *Bacteroides* [414].

In the pathological conditions of obesity and T2D, specific molecular models associated with microbes, such as LPS, play an important role in the onset of disorders associated with obesity [415,416]. LPS is a component of the cell wall of Gram-negative bacteria released by the microbiota [417]. It is an amphiphilic molecule; the hydrophilic lipid portion is associated with immunomodulation and toxicity of LPS [418]. An increased concentration of endotoxin in the blood is referred to as metabolic endotoxaemia in obese individuals [419]. In addition, the *Firmicutes:Bacteroidetes* ratio is found to be higher in very obese individuals than in “healthy obese” and lean individuals [411,420].

In the lipid storage mechanism, the abnormal expansion of white adipose mass, through hyperplasia and adipocyte hypertrophy, results in cellular stress and a local inflammatory response with the infiltration of macrophages and release of inflammatory pro-cytokines [99,421]. The low-grade chronic inflammation of adipose tissue contributes to obesity, IR, the development of hyperglycaemia, and, therefore, the manifestation of T2D [102,137].

LPS affects the secretion of pro-inflammatory cytokines and is identified as a trigger of IR [415]. LPS is able to trigger low-grade inflammation and IR when it translocates in the bloodstream [422,423,424]. The immune response occurs by binding LPS to the LPS binding protein, which activates the CD14 receptor [425]. This complex binds to toll-like receptor 4 (TLR4) on macrophages and adipose tissue, which activates the expression of genes encoding pro-inflammatory proteins, such as factor nuclear kappa B (NF-κB) and activator protein 1 (AP-1) [334,425], resulting in metabolic endotoxaemia (i.e., increased plasma LPS levels), which characterises both obesity and diabetes [415,426,427].

Intestinal bacteria can also induce metabolic endotoxaemia through the alteration and the permeability of the intestinal barrier due to the reduction of soluble IgA and the level of glucagon-like peptide-2 (GLP-2) [428,429], which improves the barrier function of the mucosa by increasing the rate proliferation of crypt cells and elongation of villus, and reducing apoptosis [429,430]. In addition, increased intestinal permeability can also derive from the reduced integrity of the epithelial tight-junction proteins (zona occludens-1 and occludin) of the intestine [366], and from the reduced thickness of the mucus layer [290] as well as from the activation of the endocannabinoid (ECB) system, which may lead to higher levels of plasma LPS [415,431].

NASH and NAFLD related to endotoxaemia are associated with greater intestinal permeability [432,433]. Increases in LPS levels and functional alterations of the intestinal barrier have been related to body mass index and high-fat diets in humans [434,435,436,437].

Dysfunction of the intestinal barrier linked to dysbiosis promotes intestinal diseases [438,439], while numerous immune disorders can result from commensal dysbiosis [327,440], such as inflammatory bowel disease (IBD) [441], rheumatoid arthritis [442], cardiometabolic diseases [443] and cancer [444].

## 4. Metabolic Syndrome and Cardiovascular Diseases

In recent decades, abundant evidence has shown that MS plays an important role in cardiovascular diseases (CVDs) [445,446].

Chronic stress is included among the main factors increasing the risk of CVD [446,447]. In the presence of oxidative stress and superoxide anions, factors of vascular homeostasis decrease and factors of contraction, such as the contraction factor derived from endothelium (EDCF), prostaglandin (PGH2), endothelin-1 (ET-1) and thromboxane A2 (TXA2), increase. O_2_^•¯^ decreases the bioavailability of NO, forms peroxynitrites and inhibits the activity of soluble guanylate cyclase (sGC) [448]. A high concentration of peroxinitrites inhibits sGC, prostacyclin production and SOD. The toxic effects of peroxynitrites on vasculature induce damage to the myocardium [449]. The loss of NO availability promotes many disorders, such as the formation of a thrombogenic surface in the vessels, an increase in the permeability of endothelium and an accumulation of oxy-LDL. This attracts monocytes and T lymphocytes, promotes the proliferation of smooth muscle cells and results in the growth of vascular walls, ultimately leading to atherosclerosis and vasculopathy [450].

Genetic and environmental factors affect the interactions between lipids, the endothelium, smooth muscle cells, inflammatory cells and the coagulation system, leading to the development of CVD.

### 4.1. Dyslipidaemia

The dyslipidaemia, an important factor in MS and defined as a common co-morbidity of diabetes and obesity, can lead to cardiovascular complications. Recent studies indicate that obese subjects exhibit concentric left ventricular hypertrophy and mild diastolic and/or systolic dysfunction [451]. Dyslipidaemia is characterised by a high plasma concentration of TG, reduced HDL and increased LDL and apolipoprotein levels [452]. The increased flow of free fatty acids into the bloodstream, resulting from IR or dysfunctional adipose tissue, is absorbed by the liver, stimulating the synthesis of triglycerides and the production of apolipoproteins B100 and VLDL [452,453]. At high levels of triglycerides, the cholesteryl ester transfer protein (CETP) promotes the exchange of a cholesterol ester from a particle of LDL or HDL with a triglyceride of a particle of VLDL [454]. The particles of LDL and HDL enriched with triglycerides are affected by hepatic lipase, which leads to the generation of particles of LDL that are both smaller and denser and particles of HDL that are smaller [455].

HDL has an inverse correlation with the risk of CVD, which includes antioxidant, anti-inflammatory, anti-thrombotic and pro-fibrinolytic activities, which contribute to its antiatherogenic role.

Different studies show that high levels of HDL are positively correlated with right ventricle functionality and pulmonary arterial hypertension [456], while high levels of LDLs are associated with increased mortality [457]. The small, dense particles of LDL are associated with an increase in cardiovascular risk. Different mechanisms may increase the atherogenicity of small dense particles of LDL, which can more quickly cross the arterial wall, bind more easily to proteoglycans in the wall of the vessel and have a greater provision to oxidation [458,459]. The circulating LDL is oxidised through the interaction with free radicals and becomes ox-LDL [460]. Ox-LDLs are one of the harmful lipids that can alter vascular functions. The development of atherosclerotic plaque is triggered by the binding of ox-LDL to a lectin-like ox-LDL receptor-1 (LOX-1) of endothelial cells.

### 4.2. Hypertension

The link between MS, hypertension and CVD is not well defined. However, abnormal metabolic pathways involved in MS can affect the cardiovascular system. Animal studies have shown that hypertension is associated with oxidative stress and the inactivation of nitric oxide production, which may cause endothelial dysfunction [461]. The vascular alterations are particularly associated with MS [462]. High blood pressure may increase arterial stiffness, and individuals with MS tend to have low capillary density [463]. Moreover, endothelial dysfunction [464] and inflammation [465] can contribute to hypertension. In patients with hypertension, the rigidity of the large elastic arteries, such as the carotids and the aorta, is accelerated [466]. Vascular degeneration causes deficient circulation, which leads to hypoxia in target organs such as the brain [467]. Endothelial dysfunction of small cerebral vessels [467,468] may lead to vascular cognitive deterioration and dementia [469].

### 4.3. Hyperglycaemia and Vascular Complications

Metabolic syndrome affects vascular complications, as observed in the mesenteric artery, heart, brain, kidneys and retina [470], as well as in pulmonary vascular function [471]. Lack of insulin secretion in T1D and a combination of IR with an inadequate compensatory response to insulin secretion in T2D lead to hyperglycaemia [472] and, consequently, to macro and microvascular diseases [470].

The high production of ROS/RNS caused by hyperglycaemia affects CVD and atherosclerosis [473,474], with vascular complications in endothelial cells (EC), smooth muscle cells (SMC) and monocytes. Endothelial cells control the spread of oxygen and carbon dioxide, revascularisation by stimulation of the vascular endothelial growth factor (VEGF), the self-regulation of blood flow, the migration of circulating cells and oversised solutes [467,475]. The endothelium increases or decreases the diameter of vessels and distal blood flow through the vasodilator mechanisms of nitric oxide, which determine the relaxation of SMC, allowing for the dilation of the vessels.

Vascular SMCs increase contractility by increasing Ca^2+^ influx and EDCF production [476]. Dysfunctional ECs cause a reduction in endothelial relaxation and increased vasoconstriction due to the reduced availability of nitric oxide [475] and increased production of contraction factors, such as endotheline-1 (ET-1) and thromboxane A2 [477,478]. In addition, the proliferation of SMC on the abluminal side [479] and platelet adhesion on the luminal side [480] restrict the vessels, while more proliferative SMC may result in increased vascular thickness [481].

EC dysfunction includes increased pro-inflammatory stimuli, adhesion molecules and reduction of barrier permeability, with infiltration of macrophages, LDL transport and production of foam cells [482,483,484]. Then, the proliferation of SMC and migration increase infiltration of inflammatory cells, the degradation of the matrix leading to the generation of plaque. Atheroma plaque develops very gradually, with enrichment in lipids, smooth muscle cells, collagen, proteoglycans and calcium [485]. The expanding atheroma swells within the arteries reducing the area of the vessel and blood flow. At the centre of an atheroma plaque, there is a lipid pool, surrounded by a fibrous cap of smooth muscle cells and a matrix rich in collagen. The production of connective tissue by fibroblasts and the deposit of calcium in the lesion cause sclerosis or hardening of the arteries; plaque rupture, formation of clots and thrombosis cause vascular occlusion and obstruction of blood flow [486,487,488]. It is estimated that about 75% of plaques that cause acute coronary incidents exhibit rupture, and about 25% show endothelial coating erosion; a limited number show calcified nodules [489]. Plaque rupture, which causes acute cardiovascular accidents, is more associated with plaques with necrotic nuclei and thinner fibrous caps, a reduced number of smooth muscle cells, a larger lipid pool and more inflammatory cells [488,490,491]. Unlike heart failure associated with atherosclerotic disease, diastolic dysfunction is a dominant aspect of the obesity-associated impairment in myocardial function [492].

## 5. Metabolic Syndrome and Neurological Diseases

Metabolic syndrome is a risk factor for neurological disorders. The brain is the most vulnerable part of the body due to high oxygen consumption and enrichment in PUFA. The brain consumes 20% more oxygen than other parts of the body.

Lipids are rich in the brain, such as cholesterol, glycerophospholipids (GP) and sphingolipids [493], and they are found to a greater extent in the plasma membrane, where they act as a barrier [494]; in nerve cells, lipids account for 50–60% of cell membrane constituents. Fatty acids are structural components of neuronal membranes. PUFAs are constituents of phospholipids and sphingolipids of cell membranes. The neuronal membrane is composed of about 50% of PUFA, while in the myelin scabbard, these lipids make up about 70% [495]. Notably, docosahexaenoic acid (DHA) and arachidonic acid (AA) are important for brain development, synaptogenesis and neurogenesis. Because brain cell membranes are rich in PUFA, they are more vulnerable to ROS/RNS, which cause oxidative stress and are prone to lipid peroxidation [496,497]. This, in turn, reduces membrane fluidity and increases membrane damage. ROS causes a harmful effect on neurons and accumulates in the brain, causing neurodegenerative diseases. Then, a reduced level of antioxidant GSH and high level of ROS in the brain lead to neurodegenerative disorders, such as Alzheimer’s disease (AD) [498], Parkinson’s disease (PD) [499], Huntington’s disease [500] and Machado–Joseph’s disease [501]. The bases of DNA/ RNA, in particular, guanine, are susceptible to oxidation with the consequent formation of 8-hydroxyquine and 8-hydroxy-2-deoxyguanosine. Carbonylation and protein nitrification are predominantly observed in the brains of patients with Alzheimer’s disease [502].

Increasing data indicate that lipid homeostasis in the nervous system is impaired during ageing and in various neurodegenerative diseases, such as AD and PD [503]. Cell membranes contain microstructures called lipid rafts, domains ordered for fluids rich in sphingolipid and cholesterol. Lipid rafts include protein complexes that interact to develop the signal regulation of the signal transduction cascade [504]. An alteration in lipid rafts can affect the amyloidogenic process and the aggregation of Aβ peptide and α-synuleic [505,506], contributing to promoting neuropathological processes as observed in AD and PD [506,507].

To explain the complex relationship between metabolic and cognitive disorders [508], a metabolic–cognitive syndrome has been proposed that considers metabolic alterations to be a continuum leading to varying degrees of cognitive disorders [509]. High levels and interactions between lipid mediators derived from phospholipids, sphingolipids and cholesterol trigger oxidative stress, neuroinflammation and apoptotic cell death [17]. In animal models, a chronic reduction in insulin receptors in the ventromedial hypothalamus produces glucose intolerance. Studies have linked IR [510], T2D, visceral obesity [511] and MS [512] to cerebral atrophy and cognitive impairment with the decline of executive function [513].

Many data show that hypertension [514], diabetes [515] and obesity [516] are linked to cardiovascular diseases, as well as to stroke, AD and depression [17]. Mechanisms may involve IR, insulin receptor and insulin growth factor (IGF) alteration, glucose toxicity, the generation of ROS and advanced glycation end products (AGE) and receptor activation for advanced glycation end products (RAGE), together with the manifestation of adipokines/cytokines and the increase in lipid mediators associated with low-grade chronic inflammation induction in the vascular and nervous systems [17]. The neurovascular unit maintains brain homeostasis [517], and its dysfunction plays a determining role regarding the onset of neurodegenerative conditions, such as stroke AD and depression [518,519].

### 5.1. Stroke

MS is the link between cardiovascular and cerebrovascular diseases, including cerebral infarction. In fact, an alteration of antioxidant systems, an increase in lipid peroxidation products and an inflammatory state have been observed in patients with stroke [520]. IR/hyperinsulinaemia, proven risks of hypertension, dyslipidaemia and obesity increase the risk of stroke in patients with diabetes and MS [521], as IR and sympathetic nerve hyperactivity can raise blood pressure, causing spontaneous intracerebral haemorrhages [522]. High glycaemia is a strong predictive cause of recurrent stroke, especially among women compared to men, and with ischemic stroke [523]. The phenomenon is associated with the increased presence of endothelial dysfunction and hypertension in diabetic women and the inflammatory effects of diabetes on the increased damage of stroke to the brain [524]. IR and hyperinsulinaemia can explain the increased risk of stroke in people with diabetes and MS [525]. Stroke can trigger neurodegeneration and cognitive decline [526], possibly inducing inflammation [527]. Regarding the incidence and risk factors associated with pre- and post-stroke dementia, the mechanisms remain unclear [528], beyond the fact that neurodegenerative and vascular mechanisms contribute to cognitive decline. The examination of the incidence of stroke and dementia for 12 years [529,530] suggests that the prevention of stroke can also prevent certain forms of dementia.

### 5.2. Alzheimer

Alzheimer’s disease is the most common form of dementia in old age. About 44 million people live with dementia in the world, and about 70% is caused by AD, with a great impact on both health and social systems [531]. In Europe, dementia affected about 10.5 million citizens between 30 and 95+ years of age in 2015 and is estimated to increase to 13.42 million people by 2030 [532]. AD is characterised by memory loss and cognitive decline, also associated with behavioural disorders [533,534]. The exact cause of AD is not yet known, but environmental factors and hereditary predisposition may contribute to its onset. Alzheimer’s disease is manifested by the deposition of protein aggregates, including extracellular amyloid plaques (Aβ), hyperphosphorylation of the Tau protein, formation of neurofibrillary tangles and loss of synapses and neurons, responsible for cerebral atrophy and cognitive decline [535,536,537]. In particular, the aggregation of Aβ results from an abnormal splitting of the amyloid precursor protein by β- and γ-secretases to produce Aβ peptides with amino acid residues [538]; diffuse plaques are the result of the deposition of both Aβ-42 and Aβ-40 [539]. The extracellular transport of Aβ is accelerated by insulin, thus explaining why diabetics have a significantly increased incidence of AD [540]. Although the Aβ peptides have a role in the defence of the brain from infections [111], their accumulation promotes an inflammatory response mediated by microglia and astrocytes [541]. Whether the inflammation is the cause or the consequence of the accumulation of Aβ is not yet clear. However, an important role of the immune system has been proposed in the development or progression of AD [542,543].

Neuroinflammatory responses involve both cellular and molecular players [544] and are also based on the activation of NLRP3 microglial inflammasome. Aβ deposits have been shown to activate NLRP inflammasome, leading to the production of IL-1β and IL-18, which can contribute to the pathogenesis of AD and cause cognitive impairment [545,546]. The onset of neurodegeneration in AD commonly occurs after age 65. However, an early form of AD has also been reported before the age of 65 due to genetic mutations that lead to an overproduction of amyloid Aβ peptides in the brain of the patient. In both forms of AD, Aβ cascades have been involved in neuronal loss, memory loss and alterations of other cognitive functions [547]. Systemic inflammation impairs the blood–brain barrier, which becomes more prone to altered secretory transport and functioning, and which leads to neurodegenerative AD disorders [548]. Obesity and diabetes are characterised by a broken blood–brain barrier with noticeably lower levels of annexe A1 expression [549].

### 5.3. Depression

Metabolic syndrome can lead to depression in young people, adults [550] and middle-aged people [551], causing disability and economic damage [552].

The hippocampus represents the key brain area for the mediation of cognitive disorders linked to MS associated with emotional alterations. Chronic stress is one of the main contributors to the development of depression due to the deregulation of the hypothalamus–pituitary–adrenal axis (HPA) and the autonomic nervous system. Stress stimulates the rapid release of cortisol and noradrenaline.

Among the biological constituents that can affect brain activity, insulin, leptin and inflammatory factors are the basis of behavioural alterations associated with MS [512].

Different studies and meta-analyses have shown that depressive and anxiety disorders are linked to physiological disorders such as systemic inflammation [553], oxidative stress [554], hyperactivity of the hypothalamic–pituitary–adrenal axis (HPA) [555] and dysregulated autonomous tone [556], along with an alteration of MS [552]. Meta-analyses indicate that people with depressive and anxiety disorders have higher risks of diabetes, stroke, obesity [557], physical decline and cognitive decline [558]. Type 2 diabetes and depression are linked together through stress that alters the brain’s ability to regulate the release of corticosteroids, resulting in hypercortisolaemia [559]. Excessive stimulation of corticosteroid receptors in the hippocampus can lead to atrophy of the hippocampus, causing depression and dementia [560]. Depression is related to obesity through the disordering of the autonomic nervous system and HPA [561].

## 6. Management of Metabolic Syndrome

The modern management of MS involves a multidisciplinary approach that combines lifestyle changes and pharmacological interventions. Pharmacotherapy and associated comorbidities necessitate the prolonged use of multiple medications, which is challenging for patients with poor compliance. Thus, there is a growing interest in lifestyle changes to the management of metabolic dysfunction, such as the control of body weight and healthy diets.

Based on animal and human studies, anti-oxidative therapies have been found to be effective in the treatment of a common node, such as redox imbalance, between multifactorial disorders associated with MS [562,563]. The imbalance between free radicals/oxidants and antioxidant defences leads to oxidative stress, which promotes a wide range of clinical disorders, both as a source and as a result, and diseases [31].

Antioxidant systems include endogenous antioxidant defence mechanisms that act along with exogenous antioxidants, such as vitamins and derivatives of dietary polyphenols, to counteract stress and oxidative damage [564]. Antioxidants, such as vitamins (E, C, Q) and carotenoids or polyphenols (as phenolic acids and flavonoids), are derived from food [565,566,567]. Antioxidants act synergistically by trapping single electrons from free radicals or by reducing ROS enzymatically. There is a general trend toward the use of natural rather than synthetic antioxidants [568,569]. Plant antioxidant therapies have shown significant effects in various stress conditions [561,562,570,571] and in the protection of diseases associated with MS [572]. Many natural compounds derived from plant extracts, spices, herbs and essential oils have beneficial effects in patients with MS [573,574,575]. Polyphenols are the most prevalent antioxidants in plant-based diets, including fruits, vegetables and cereals [576], whose consumption reduces the risk of MS [337]. A high-quality plant-based diet is an effective intervention for weight management [577]. A higher consumption of fruits and vegetables reduces the risk of cognitive impairment and dementia [578]. The phytochemicals of fruits and vegetables have protective effects against PD [579]. Mediterranean diets rich in neuroprotective nutrients have a beneficial effect on developing Alzheimer’s disease [580]. However, many problems still remain elusive: most exogenously administrated antioxidants are not selective or uniformly distributed in the various parts of cells or tissues [581,582]; the threshold level of antioxidant nutrients needed for optimal nutrition is unclear [573,583], as well as the specificity of antioxidants and their possible interactions [573,584]. Therefore, it is suggested to focus on developing innovative targeted antioxidants to achieve precise therapeutic effects [581,585].

Lifestyle is important in the prevention and treatment of obesity, diabetes and diseases linked to MS [586]. Weight loss may prevent and reverse diabetes [587,588,589] and improve blood glucose, insulin sensitivity and comorbidities [590]. The decrease in weight can reduce cardiovascular risk associated with obesity and diabetes [591,592]. Dietary energy restriction promotes weight loss and reduces risks of metabolic disorders [593,594]. It also improves lipid and cytokine profiles, reduces cardiovascular risks [595] and improves blood sugar and insulin sensitivity in obese patients with T2D [596]. Johnston and coworkers [597] reported that low carbohydrate ketogenic diets were similarly effective in reducing body weight and IR in patients with diabesity. Dietary protein restriction has been associated with a reduction in diabetes [593] and can lead to the same clinical results as calorie restriction without reducing calorie intake [598]. Evidence indicates that intermittent fasting can replace the mechanisms of dietary or caloric restriction in weight loss [599,600].

Microbiota control can play an important role in the development of obesity and diabetes [601]. The Mediterranean diet is associated with a wide range of benefits in young and adult patients with diabesity and metabolic syndrome in the prevention of derived complications [602,603,604,605,606], partly due to the ability to regulate microbial populations, improving the growth of *Lactobacillus* spp., *Bifidobacterium* spp. and *Prevotella* spp. and limiting *Clostridium* spp. development [607]. Restoring intestinal microbiota composition and function can have a significant impact on improving cardiovascular disease [608] and neurodegenerative diseases [609].

## 7. Conclusions and Future Perspectives

MS is a growing disorder that affects thousands of people around the world, especially in industrialised countries, increasing mortality.

Hyperglycaemia, IR, inflammation, oxidative stress, dysbiosis, abdominal obesity, atherogenic dyslipidaemia and hypertension are the main pathological comorbidities associated with MS. All cellular and biochemical alterations observed in MS as dysregulation in the glucose and lipid metabolism, expression in immune response, impairment of endothelial cell function and dysbiosis may represent a pathological bridge between MS and diseases. These factors, taken together, constitute the best indicator of the MS risk for diabesity, cardiovascular diseases and neurological disorders. Recent discoveries improve our understanding of them and could lead to better therapeutic strategies in the future. The interaction between the microbiome, metabolic processes and health outcomes should also be considered relevant to these health effects, justifying further research. A better understanding of metabolic disorders is expected to promote the development of new biomarkers for risk or diagnosis, as well as beneficial treatments to reduce diseases associated with metabolic syndrome, including integrative approaches aimed at improving lifestyle and diet routine.

## Figures and Tables

**Table 1:** Diagnostic clinical criteria of metabolic syndrome by different health organizations.

Clinical Parameters	Criteria					
	Central Obesity	Fasting Blood Sugar	↑ Triglycerides	↓ HDL-Cholesterol	↑ Blood Pressure	Diagnosed as MS
IDF (2005) [19]	Waist circumference defined in terms of ethnicity-specific values	≥100 mg/dL or on medication	≥150 mg/dL or on medication	Male: <40 mg/dL Female: <50 mg/dL	Diastolic ≥130 and/or systolic ≥85 mmHg or on medication	Absolutely required central obesity plus ≥2 criteria
AHA/NHLBI (2005) [20]	Waist circumference Male: ≥102 cm Female: ≥88 cm	≥100 mg/dL or on medication	≥150 mg/dL or on medication	Male: <40 mg/dL Female: <50 mg/dL	Diastolic ≥130 and/or systolic ≥85 mmHg or on medication	≥3 criteria
AHA/NHLBI and IDF:2009 [21]	Waist circumference defined in terms of population- and country-based-specific definition	≥100 mg/dL or on medication	≥150 mg/dL or on medication	Male: <40 mg/dL Female: <50 mg/dL	Diastolic ≥130 and/or systolic ≥85 mmHg or on medication	≥3 criteria

Note: IDF, International Diabetes Federation. AHA/NHLBI, American Heart Association/National Heart, Lung and Blood Institute.

## Data Availability

No data were used for the research described in this article.
